# Acute Cholecystitis Complicating Cardiac Disease: A Cohort Study From a Tertiary Care Center in Mexico City, Mexico

**DOI:** 10.7759/cureus.53915

**Published:** 2024-02-09

**Authors:** Fernando Alonso Núñez Moreno, Vanessa Ortiz Higareda, Luis León Hernández Trejo, Lissvia Estéfani Acosta Gaxiola

**Affiliations:** 1 Gastrointestinal Surgery, Hospital de Especialidades, UMAE Centro Médico Nacional Siglo XXI, Mexico City, MEX; 2 Cardiothoracic Surgery, Hospital de Cardiologia, UMAE Centro Médico Nacional Siglo XXI, Mexico City, MEX; 3 General Practice, Hospital General Regional No. 1, Instituto Mexicano del Seguro Social (IMSS), Tijuana, MEX

**Keywords:** necrotic gallbladder, lap chole, heart disease, heart surgery, acalculous gallbladder

## Abstract

Background

Acute cholecystitis (AC) presents as inflammation of the gallbladder, predominantly attributed to gallstones obstructing the cystic duct. Another notable etiology is ischemic cholecystitis, often stemming from severe illnesses that compromise blood flow to the splanchnic system. Individuals with pre-existing cardiovascular conditions or undergoing cardiopulmonary surgery encounter elevated risks of gastrointestinal pathology, leading to heightened morbidity and mortality rates. In these cases, AC stands out as a significant concern, whether it originates from gallstones or is acalculous (ischemic).

Methods

We conducted a single-center, retrospective cohort study at the National Medical Center in Mexico City, Mexico. We included demographic, clinical, laboratory, preoperative, intraoperative, and postoperative data. Our main objectives were to describe the characteristics of our population and identify predictors of major complications following surgery for AC in patients with heart disease.

Results

Our study encompassed 18 patients diagnosed with both AC and cardiac disease, with acute myocardial infarction being the leading cause of admission, constituting 38.9% of cases. The clinical presentation was characterized by right hypochondrial pain, observed in all patients, while Murphy's sign was evident in 44.4% of cases. Additionally, various non-specific symptoms were present in varying percentages across the patient cohort.

Concerning imaging studies, 88% of our cohort underwent ultrasound (USG), while computed tomography (CT) was utilized in 55% of patients. Among those who had USG, the most prevalent findings were gallbladder wall thickening in 56% and perivesicular fluid in 37.5%. In patients who underwent CT scans, the predominant observations included perivesicular fluid in 66% and perivesicular fat stranding in 55%. In terms of intraoperative findings, gallbladder empyema and necrosis were the most common findings identified in 55.6% of the cases.

After surgery, 61.1% had no complications or developed mild complications, while 11.1% experienced moderate to severe complications without resulting in death; the mortality rate was 27.8% in our cohort. Overall, major complications or death were present in 38.9% of our cohort. In the postoperative data analysis, the use of mechanical ventilation and vasopressors showed a significant correlation with increased morbidity and mortality.

Conclusions

Our study contributes valuable information to the international literature, providing insights into the complications faced by the Mexican population in the context of heart diseases, particularly among patients suffering from cholecystitis. Within individuals with cardiac disease, the occurrence of AC requiring surgical intervention is associated with heightened morbidity and mortality rates, with our cohort experiencing rates as high as 38.9%. Consistent with findings in the international literature, these results underscore the critical importance of continuing the search for novel clinical or laboratory predictors for this high-risk population. While most parameters assessed in this study did not show any correlation with major complications, exceptions were observed in postoperative vasopressors and mechanical ventilation.

## Introduction

Acute cholecystitis (AC) presents as inflammation of the gallbladder, predominantly attributed to gallstones obstructing the cystic duct. Another notable etiology is ischemic cholecystitis, often stemming from severe illnesses that compromise blood flow to the splanchnic system [1-6]. The complexity of AC poses an additional threat to individuals already grappling with severe diseases, augmenting the risk of mortality. This complication has been notably observed in patients within the intensive care unit, in individuals with heart disease, or those who have undergone cardiac surgery [6-8].

Diagnosing AC in patients dealing with severe diseases presents a significant challenge, exacerbated by a notable absence of predictors - whether clinical, biochemical, or radiological tests - with the necessary efficacy to discern high-risk patients [9,10]. The incidence of this complication in patients undergoing heart surgery has been reported at 0.11%, with variable mortality [7]. However, there is a lack of information regarding this type of complication in the Latin American population. This study addresses a critical gap by investigating a previously unstudied population, specifically within a tertiary care center for individuals with heart diseases in the Mexican population. In this retrospective study, we aim to to identify predictors, whether they be clinical or biochemical, of major complications and mortality, and to delineate the distinctive characteristics defining this particular patient cohort.

## Materials and methods

Data collection

We conducted a retrospective analysis of the medical records of patients hospitalized at UMAE Centro Médico Nacional Siglo XXI (National Medical Center), Mexico City, Mexico, comprising two hospitals: the Specialties Hospital and the Cardiology Hospital.

The data analysis involved patients admitted between January 2017 and July 2022, either for cardiac pathology or those undergoing cardiopulmonary surgery. Specifically, our focus was on individuals who developed AC as a complication during their hospitalization and subsequently underwent cholecystectomy within the same period. During this period, 42 records were identified, but only 18 of them met the inclusion criteria. All procedures were performed by the same group of surgeons from the Gastrointestinal Surgery department at the Specialties Hospital.

Data were meticulously collected from various sources, including admission notes, clinical records, preoperative and operative documentation, anesthetic records, nursing logs, laboratory reports, imaging findings, and death certificates extracted from comprehensive medical records. Subsequently, this wealth of information was organized and stored in an SQLite3 database. The data management process was facilitated using the Python programming language, with Visual Code as the code editor and Streamlit as the graphical interface [11,12].

Exclusion criteria included patients with incomplete medical data, those not undergoing cholecystectomy, individuals under the age of 18, and patients with other acute gastrointestinal complications (acute pancreatitis, mesenteric ischemia, volvulus, etc.).

The diagnosis of AC was made by attending surgeons based on clinical, laboratory, and radiological data. The clinical diagnosis was confirmed with surgical findings and pathology reports.

Data analysis

For the data analysis, we employed R and Python libraries such as Matplotlib, Pandas, Stats, and NumPy. These tools were used to calculate measures of central tendency, ranges, and other descriptive statistics.

Demographic data was analyzed, and the mean age, BMI, and height were obtained. American Society of Anesthesiologists (ASA) classification was derived from medical records established by the anesthesiologist on the day of surgery, and severity classification was conducted following TG18 guidelines. Complications were categorized using the Clavien-Dindo system for postoperative complications, where minor complications correspond to CD < II, major complications to CD III and IV, and CD V designates patients who succumbed. The cause of death was retrieved from official death records compiled by the attending surgeon.

For the paired analysis, clinical and laboratory data from patients who experienced major complications (CD > III) were compared between the time of admission and up to 24 hours before surgery. A Shapiro-Wilk test was conducted to determine the parametric or non-parametric nature of the data. A paired t-test was performed using the Python Stats library on data suitable for parametric analysis, while a Wilcoxon signed-rank test was applied to data with non-parametric values.

An analysis of preoperative data with the Kruskall-Wallis test was performed to detect differences among the following groups: The group with minor complications (CD < II), group with major complications (CD III and IV), and group with major complications and patients that died (>CD III). No post hoc analysis was performed due to the lack of statistical difference among the groups.

For the analysis of factors potentially associated with major complications, we formed two groups: one with minor complications (CD < II and no complications), and one with major complications and patients who died (CD > III). For quantitative data, we used the Mann-Whitney U and non-paired Student's t-test, and for qualitative data, we employed Fisher's exact test. P-values < 0.05 were considered statistically significant.

Ethical approval for this study was obtained in July 2022 from both the Investigation and Ethics Committee of our institution. Individual patient consent was not obtained since the study was approved as a retrospective cohort.

## Results

Baseline characteristics and medical history

Out of the total patient cohort, 10 (56%) were male, while 8 (44%) were female. The average age of the participants was 67 years, with a standard deviation of ±11.4, a median of 66 years, and an age range spanning from 39 to 83 years. Among the patients, 44.4% were identified as overweight, with 11% exhibiting obesity grades I and II.

For surgical risk assessment, the ASA classification was employed, showing ASA II at 11.1%, ASA III at 22.2%, and ASA IV at 66.7%. To assess the severity of AC, we classified patients according to the Tokyo guidelines criteria (TG-18), as having a mild, moderate, and severe disease; mild disease was found in 27.8%, moderate disease in 55.6%, and severe disease in 16.7%.

Tobacco usage was prevalent in 50% of the population, averaging 17.33 packs per year (SD 19.5). A range of comorbidities were identified, including systemic arterial hypertension in 61.1%, acute myocardial infarction in 44.4%, diabetes mellitus in 38.9%, valvulopathy in 22.2%, atrial fibrillation in 22.2%, heart failure in 11%, dilated cardiomyopathy in 5.6%, atrioventricular block in 5.6%, and dyslipidemias in 11.1%. As expected systemic arterial hypertension and a history of acute myocardial infarction emerged as the most prevalent conditions within this cohort (see Table 1).

**Table 1:** Baseline characteristics and medical history TG18: Tokyo guidelines 2018; BMI: Body mass index; ASA: American Society of Anesthesiologists *Mean values are displayed

Baseline characteristics	n	%
Age *	67	-
Gender (male/female)	10/8	56/44
Weight (kg) *	70.89	-
Height (m) *	1.637	-
BMI *	26.4	-
Obesity and malnutrition		
-Malnutrition	0	-
-Normal	8	44.4
-Overweight	8	44.4
-Grade I obesity	1	5.5
-Grade II obesity	1	5.5
-Grade III obesity	0	-
Preoperative assessment		
ASA I	0	0.0
ASA II	2	11.1
ASA III	4	22.2
ASA IV	12	66.7
ASA V	0	0.0
ASA VI	0	0.0
Cholecystitis severity TG18		
Tokyo I	5	27.8
Tokyo II	10	55.6
Tokyo III	3	16.7
Medical history		
Smoking	9	50.0
Arterial hypertension	11	61.1
History of acute myocardial infarction	8	44.4
Diabetes mellitus	7	38.9
Valvulopathy	4	22.2
Atrial fibrillation	4	22.2
Heart failure	2	11.1
Dyslipidemia	2	11.1
Dilated cardiomyopathy	1	5.6
Atrioventricular block	1	5.6
Others (venous insufficiency, hypothyroidism, etc.)	6	33.3

Reason for admission 

The admission reasons were diverse, with 38.9% of patients admitted for acute myocardial infarction, 16.6% scheduled for cardiovascular surgery, 11.1% presenting with unstable angina, 11.1% undergoing pacemaker placement, another 11.1% requiring urgent cardiac catheterization, 5.6% undergoing valve replacement, and the remaining 11.1% admitted due to heart failure (see Table 2).

**Table 2:** Reason for hospital admission

Reason for admission	n	%
Acute myocardial Infarction	7	38.9
Scheduled cardiovascular surgery	3	16.6
Unstable angina	2	11.1
Emergency pacemaker placement	2	11.1
Emergency cardiac catheterization	2	11.1
Heart failure	2	11.1

Clinical presentation

The most common symptom was right hypochondrial pain, this symptom was present in all patients, 44.4% showed Murphy's sign, 33.3% experienced nausea, 22.2% fever, and a similar percentage reported diffuse abdominal pain. Jaundice was observed in 5.6% of cases (see Table 3).

**Table 3:** Clinical presentation

Signs and symptoms	n	%
Right hypochondrial pain	18	100
Murphy's sign	8	44.4
Nausea	6	33.3
Fever	4	22.2
Diffuse abdominal pain	4	22.2
Jaundice	1	5.6

Imaging studies 

Ultrasound (USG) was conducted in 88% of the patients, while computed tomography (CT) was performed in 50% of cases, with both modalities being used in combination in 38% of instances. Magnetic resonance imaging or scintigraphy was not employed in this patient cohort.

Among the patients who underwent USG (88%), the most prevalent findings suggestive of AC included gallbladder wall thickening noted in 56% of patients, gallbladder sludge and gallstones in 50%, perivesicular fluid in 37.5%, and hydrops of the gallbladder in 6.25%.

For patients who had a CT scan requisitioned (50%), prevalent findings included perivesicular fluid in 66%, fat stranding in 55%, hydrops of the gallbladder in 55%, gallbladder wall thickening in 44%, gallstones in 33%, gallbladder wall enhancement in 11%, and choledocholithiasis in 11% (Table 4).

**Table 4:** Abdominal ultrasound and computed tomography findings -: USG findings; *: CT findings

Imaging studies	n	%
Abdominal ultrasound	16	88
-Thickening of the gallbladder wall	9	56
-Gallstones	8	50
-Gallbladder sludge	8	50
-Perivesicular fluid	6	37.5
-Hydrops of the gallbladder	1	6.25
Computed tomography	9	50
*Perivesicular fluid	6	66
*Perivesicular fat stranding	5	55
*Hydrops of the gallbladder	5	55
*Thickening of the gallbladder wall	4	44
*Gallstones	3	33
*Gallbladder wall contrast enhancement	1	11
*Choledocholithiasis	1	11

Data on surgical interventions

Concerning the surgical data, cholecystectomy was performed as an emergency procedure in 83.3% of cases, with the remaining 16.7% being elective surgeries. An open approach was utilized in 83.3% of cases, while a laparoscopic approach was employed initially in 16.7%, with a conversion rate to an open approach of 66.6%. The average surgical time was 109 minutes (SD 45.31). The average time from symptom onset to surgical treatment was 6 days (SD ±5.5 days), with a median of 4.5 days, and a range from 1 day to 24 days (see Table 5).

**Table 5:** Surgery data *: Time in days

Surgery data	n	%
Emergency surgery	15	83.3
Elective surgery	3	16.7
Open surgery	15	83.3
Laparoscopic surgery	3	16.7
-Conversion to open surgery	2	66.6
Time to surgery *	6	

Surgical findings

In all cases, surgical findings confirmed the diagnosis of complicated cholecystitis, being the most frequent findings of gallbladder necrosis in 55.6% and gallbladder empyema in 55.6%, followed by hydrops of the gallbladder in 50%, thickening of the gallbladder wall in 44.4%, gallbladder perforation in 22.2%, perivesicular fluid in 16.7%, cystic duct necrosis in 11.1%, and cystic artery thrombosis in 5.6% (see Table 6).

**Table 6:** Surgical findings

Surgical findings	n	%
Empyema of the gallbladder	10	55.6
Gallbladder necrosis	10	55.6
Hydrops of the gallbladder	9	50
Thickening of the gallbladder wall	8	44.4
Gallbladder perforation	4	22.2
Perivesicular fluid	3	16.7
Cystic duct necrosis	2	11.1
Cystic artery thrombosis	1	5.6

Complications and death

In terms of postoperative complications, 61.1% of individuals experienced either no complications or only minor issues (Clavien-Dindo < II), including seromas, wound infections, or postoperative pain, all of which were managed without surgical intervention. Notably, 11.2% encountered significant complications necessitating some form of intervention (Clavien-Dindo III and IV). The overall mortality rate was 27.8% (Clavien-Dindo V), resulting in a cumulative 38.9% of patients facing major complications or death (Table 7).

**Table 7:** Postoperative complications

Postoperative complications	n	%
No complications	2	11.1
Clavien-Dindo I	6	33.3
Clavien-Dindo II	3	16.7
Clavien-Dindo III	1	5.6
Clavien-Dindo IV	1	5.6
Clavien-Dindo V (mortality)	5	27.8

Cause of death

The most common cause of death was abdominal sepsis, accounting for 60% of deaths. Additionally, 20% of deaths were due to cardiac shock, and another 20% resulted from respiratory failure (see Table 8).

**Table 8:** Cause of death

Cause of death	n	%
Abdominal sepsis	3	60
Cardiogenic shock	1	20
Respiratory failure	1	20

Paired analysis of clinical and laboratory measurements during admission and pre-surgery

Upon analyzing paired laboratory and clinical values, no significant variations were observed between the admission and preoperative measurements in patients with major complications (CD > III). Hematocrit had a disparity between the measurement during admission and preoperative but this difference was not statistically meaningful. There were no differences detected in vital signs, leukocytes, RDW, platelets, and other laboratory parameters (see Table 9).

**Table 9:** Paired analysis of clinical and laboratory measurements during admission and pre-surgery CD: Clavien-Dindo classification; RDW: Red cell distribution width; AST: Aspartate aminotransferase; ALT: Alanine aminotransferase; INR: International normalized ratio

Parameters	Admission	Preoperative	p
Heart rate (beats/min)	75	75	0.184
Respiratory rate (breaths/min)	19	20	0.728
Systolic blood pressure (mmHg)	104	112	0.407
Diastolic blood pressure (mmHg)	63	71	0.453
Temperature (°C)	36.4	36.5	0.775
Leukocytes (cells/mm³)	12.8	17.3	0.130
Hematocrit (%)	42	39	0.067
RDW	15	15	0.604
Platelets (cells/mm³)	195	273	0.970
AST (mg/dL)	107	48	0.529
ALT (mg/dL)	43	47	0.489
Total bilirubin (mg/dL)	1.4	2	0.944
INR	1.40	1.34	0.522
Creatinine (mg/dL)	1.17	1.28	0.208

Comparative analysis of preoperative laboratory measurements and complications

An analysis of preoperative laboratory measurements among groups using the Kruskall-Wallis test was performed. A difference was found among groups in platelet measurements, but this difference was not statistically significant. No differences were found in the rest of the laboratory values when comparing patients with minor complications, patients with major complications, and patients who experienced major complications or death (see Table 10).

**Table 10:** Laboratory measurements comparison among groups CD: Clavien-Dindo complications classification; NC: No complications; RDW: Red cell distribution width; AST: Aspartate aminotransferase; ALT: Alanine aminotransferase; INR: International normalized ratio

Laboratory parameters	NC/CD < II	CD III/IV	CD > III	p
Leukocytes (cells/mm^3^)	13.3	14.0	17.4	0.253
RDW	16.1	14.0	15.6	0.740
Hematocrit (%)	36.9	35.0	38.6	0.857
Platelets (cells/mm^3^)	251	514	153	0.056
AST (mg/dL)	82.5	51.0	46.8	0.920
ALT (mg/dL)	217.3	28.5	54.0	0.807
Total bilirrubin (mg/dL)	1.2	3.03	1.84	0.236
INR	1.3	1.3	1.4	0.521
Creatinine (mg/dL)	1.3	1.4	2.5	0.144

Factors associated with major complications and death

There were no significant differences observed between genders, age, BMI, smoking history, and preoperative vasopressor use. Patients with minor complications had a shorter operating time, averaging 93 minutes, in comparison to those with major complications, whose surgeries lasted an average of 131 minutes. However, this difference was not statistically significant. The ASA and Tokyo classifications did not correlate with an increase in complications.

Examining reasons for admission, no substantial relationship with increased morbidity or mortality was observed. Intraoperatively, no significant differences in findings were noted. Notably, gallbladder necrosis, the most prevalent discovery, was found in 22.2% of patients with minor complications and 33.3% of those with major complications; however, this disparity did not reach statistical significance. Regarding acalculous cholecystitis, minor complications in 27.8% of patients and major complications in 22.2% with no significant difference.

A statistical difference emerged in patients requiring postoperative ventilation and postoperative vasopressors; however, this was expected due to the characteristics of the patients (Table 11).

**Table 11:** Factors associated with major complications and death NC: No complications; BMI: Body mass index; ASA: American Society of Anesthesiologists * p < 0.05

Parameters	NC/CD < II	%	CD > III	%	p
Male/female	5/6	27.8/33.3	5/2	27.8/11.1	0.367
Age	64.7	-	70.6	-	0.303
BMI	27.1	-	25.3	-	0.348
Postoperative mechanical ventilation	2	11.1	5	27.8	0.049*
Smoking history	4	22.2	5	27.8	0.334
Preoperative vasopressor	4	22.2	3	16.7	0.643
Postoperative vasopressor	2	11.1	6	33.3	0.012*
Operating time (min)	93	-	131	-	0.061
ASA score					0.464
ASA I	0	0.0	0	0.0	
ASA II	2	11.1	0	0.0	
ASA III	3	16.7	1	5.6	
ASA IV	6	33.3	6	33.3	
ASA V	0	0.0	0	0.0	
ASA VI	0	0.0	0	0.0	
Tokyo					0.170
Tokyo I	4	22.2	1	5.6	
Tokyo II	4	22.2	6	33.3	
Tokyo III	3	16.7	0	0.0	
Acute myocardial infarction	5	27.8	4	22.2	0.500
Arhythmia	0	0.0	2	11.1	0.137
Cardiovascular surgery	3	16.7	0	0.0	0.202
Congestive heart disease	1	5.6	0	0.0	0.600
Hydrops of the gallbladder	5	27.8	4	22.2	1.000
Acalculous cholecystitis	5	27.8	4	22.2	1.000
Gallbladder necrosis	4	22.2	6	33.3	0.066
Cystic duct necrosis	1	5.6	1	5.6	1.000
Perivesicular fluid	2	11.1	1	5.6	1.000
Empyema of the gallbladder	7	38.9	3	16.7	0.630
Thickened gallbladder wall	6	33.3	2	11.1	0.367
Gallbladder perforation	1	5.6	3	16.7	0.245

## Discussion

To the best of our knowledge, this study stands as the sole investigation carried out on patients presenting with both AC and cardiac diseases in Latin America.

The demographic analysis revealed a mean age of 67 years, aligning closely with findings reported by Saito et al. in Japan back in 1997 [8]. Nonetheless, the average age exhibits variations across international literature, thereby underscoring notable differences among them [8,13].

The gender distribution slightly favored males (56% men vs. 44% women), in line with prior reports in the international literature [13]. The average BMI in our study was 26.4, with 88.8% of participants categorized as normal weight or overweight, and a minority (11%) being obese. This aligns with reports of patients with cardiac disorders and critically ill patients in intensive care units who had AC, similar to our study, no association was demonstrated between overweight or obesity and an increase in the likelihood of experiencing major complications or death [10,14].

Concerning comorbidities, hypertension, a history of acute myocardial infarction, and diabetes mellitus were the most commonly found conditions in our population, consistent with international literature [8,15]. The most common reason for hospital admission was acute myocardial infarction, accounting for nearly 40% of cases, followed by scheduled cardiovascular surgery in 16.7%. The most frequently reported symptoms were right hypochondrial pain and Murphy's sign, consistent with findings previously published by Passage et al. in 2007 [7].

The interval between symptom onset and cholecystectomy averaged six days, nearly double the duration reported by Saito et al. in 1997 [8]. This discrepancy may be attributed to difficulties in correct diagnosis or hospital protocols, leading to an excessive delay in surgical treatment. In line with this, Ozeki et al. in 2015 emphasized the diagnostic complexities of cholecystitis in this special population, in their study in a specialized cardiology hospital, 16 patients initially presented with symptoms and laboratory alterations resembling acute coronary syndrome but ultimately were diagnosed with AC [10]. This underscores the difficulty in differentiating between cardiac and gastrointestinal complications, thereby delaying prompt surgical treatment. To assess whether this delay in treatment affected patients' prognosis, we examined those who underwent surgery before and after seven days from the onset of the first symptoms; no significant differences in complications were found in our cohort (unpublished data).

Various imaging studies, primarily USG and to a lesser extent CT, were employed. Gallbladder wall thickening was the most frequently mentioned USG finding, similar to Passage et al.'s findings in 2007 [7]. In CT scans, perivesicular fluid, hydrops of the gallbladder, and perivesicular fat stranding were the most frequently found signs. The ASA classification for preoperative risk and the TG18 severity classification for AC did not differ between groups experiencing major and minor complications [16].

The most frequently mentioned intraoperative findings were empyema of the gallbladder, gallbladder necrosis, hydrops of the gallbladder, and gallbladder wall thickening, present in approximately half of the patients, gallbladder perforation was found in 22.2% of the patients. These findings are similar to 2004 findings from Laurila in critically ill patients with acute acalculous cholecystitis [14].

The overall mortality rate was 27.8%, with abdominal sepsis being the most common cause of death (60%). Major complications and death occurred in 39% of the patients in our cohort, which is similar to what was reported by Passage et al. in 2007 [7]. Clinical and laboratory values at admission and preoperatively did not show statistically significant differences. The use of these parameters to predict major complications or increased mortality does not seem to be useful. Differences in pre-surgery laboratory values among patients with minor complications, major complications, and those who died were not present in the Kruskall-Wallis test. The Sequential Organ Failure Assessment (SOFA) scale could not be compared due to a lack of data. However, it has been proposed as a possible marker for major postoperative complications in this type of patient, especially when measured in the pre-surgical period [14].

Factors such as gender, age, BMI, reasons for admission, surgical time, and intraoperative findings did not exhibit statistically significant differences between patients with minor and major complications. Similarly, no significant differences were found between patients with acalculous and calculous cholecystitis regarding the prevalence of major complications. This observation aligns with previous reports in the international literature concerning patients who underwent cardiac surgery and subsequently developed gastrointestinal complications [7,17].

Regarding the main hypothesis of this study, the majority of the factors examined prove ineffective in predicting patients at a heightened risk of major complications. The exception to this lies in the utilization of mechanical ventilation and vasopressors during the postoperative phase. This observed statistical difference is attributed to the nature of the groups, where the subset experiencing major complications and patients who succumbed were significantly more likely to be in intensive care and need of supportive measures.

Given the uncommon nature of the dual association between AC and cardiac conditions, our study's scope was somewhat constrained by a relatively small number of cases observed over an extended period, despite its execution within a specialized hospital. This limitation, inherent in studying such a rare occurrence, is shared with most international publications on the subject. Our findings, though, may have relevance beyond our immediate context and could potentially apply to other Latin American countries and developing nations with similar demographics and health systems.

However, it's essential to acknowledge the retrospective nature of our study, introducing potential biases in data collection. Relying on medical records and lacking control over potential confounding factors is a common challenge in retrospective studies. Furthermore, our study includes various cardiac etiologies, akin to other internationally publicized studies, potentially limiting the extrapolation of conclusions to specific populations with distinct cardiac diseases and affecting overall generalizations.

## Conclusions

In patients with cardiac disease, the onset of AC requiring surgical intervention is associated with heightened morbidity and mortality rates. Our population appears to share similar characteristics with previous international reports, exhibiting a high morbimortality rate. Identifying predictive factors, especially those preoperative, remains crucial for recognizing individuals at an increased risk of major complications or death.

Despite the limitations of our study, it stands as one of the largest involving this patient population, constituting a noteworthy contribution. Interpretation of these conclusions should be approached with caution due to our study's limitations; however, it provides valuable insights, establishing a foundation for further research aimed at mitigating morbidity and mortality in this unique population and similar settings. The utilization of procalcitonin and the pre-surgical SOFA scale are among the newly proposed predictors that could warrant additional studies in the future. Continued exploration is needed to identify predictive factors that could enhance outcomes for these patients. It's worth noting that our hospital manages a substantial caseload, potentially positioning it as a reference center for studying this patient population.

## References

[1] Barie PS, Eachempati SR (2010). Acute acalculous cholecystitis. Gastroenterol Clin North Am.

[2] Pisano M, Allievi N, Gurusamy K (2020). 2020 World Society of emergency surgery updated guidelines for the diagnosis and treatment of acute calculus cholecystitis. World J Emerg Surg.

[3] Adachi T, Eguchi S, Muto Y (2022). Pathophysiology and pathology of acute cholecystitis: a secondary publication of the Japanese version from 1992. J Hepatobiliary Pancreat Sci.

[4] Lammert F, Gurusamy K, Ko CW (2016). Gallstones. Nat Rev Dis Primers.

[5] Chor CY, Mahmood S, Khan IH, Shirke M, Harky A (2020). Gastrointestinal complications following cardiac surgery. Asian Cardiovasc Thorac Ann.

[6] Gulkarov I, Trocciola SM, Yokoyama CC, Girardi LN, Krieger KK, Isom OW, Salemi A (2014). Gastrointestinal complications after mitral valve surgery. Ann Thorac Cardiovasc Surg.

[7] Passage J, Joshi P, Mullany DV (2007). Acute cholecystitis complicating cardiac surgery: case series involving more than 16,000 patients. Ann Thorac Surg.

[8] Saito A, Shirai Y, Ohzeki H, Hayashi J, Eguchi S (1997). Acute acalculous cholecystitis after cardiovascular surgery. Surg Today.

[9] Bagla P, Sarria JC, Riall TS (2016). Management of acute cholecystitis. Curr Opin Infect Dis.

[10] Ozeki M, Takeda Y, Morita H, Miyamura M, Sohmiya K, Hoshiga M, Ishizaka N (2015). Acute cholecystitis mimicking or accompanying cardiovascular disease among Japanese patients hospitalized in a cardiology department. BMC Res Notes.

[11] van Rossum G (1995). Python tutorial, Technical Report CS-R9526. Cent voor Wiskd en Inform.

[12] Hunter JD (2007). Matplotlib: a 2D graphics environment. Comput Sci Eng.

[13] Ganpathi IS, Diddapur RK, Eugene H, Karim M (2007). Acute acalculous cholecystitis: challenging the myths. HPB (Oxford).

[14] Laurila J, Syrjälä H, Laurila PA, Saarnio J, Ala-Kokko TI (2004). Acute acalculous cholecystitis in critically ill patients. Acta Anaesthesiol Scand.

[15] Kim SB, Gu MG, Kim KH, Kim TN (2020). Long-term outcomes of acute acalculous cholecystitis treated by non-surgical management. Medicine (Baltimore).

[16] Yokoe M, Hata J, Takada T (2018). Tokyo Guidelines 2018: diagnostic criteria and severity grading of acute cholecystitis (with videos). J Hepatobiliary Pancreat Sci.

[17] Haywood N, Mehaffey JH, Hawkins RB (2020). Gastrointestinal complications after cardiac surgery: highly morbid but improving over time. J Surg Res.

